# Clinical Application of Inferior Alveolar Nerve Block Device for Safe and Secure IANB by Any Operator

**DOI:** 10.1155/2023/1021918

**Published:** 2023-09-08

**Authors:** Tomoyasu Noguchi, Kento Odaka, Ken-ichi Fukuda

**Affiliations:** ^1^Division of Special Needs Dentistry and Orofacial Pain, Department of Oral Health and Clinical Science, Tokyo Dental College, Tokyo 101-0061, Japan; ^2^Department of Oral and Maxillofacial Radiology, Tokyo Dental College, Tokyo 101-0061, Japan

## Abstract

The inferior alveolar nerve block (IANB) is an established technique with a success rate of 60–80%; however, large errors have been reported among operators. Some dentists do not prefer to use IANB because of the risk of complications. Nevertheless, it is a useful technique for pain control, and a secure IANB offers significant benefits to operators and patients. This case series study aimed to investigate the efficacy of the “IANB Device,” a nerve block guide for IANB, and the adverse events associated with its use in clinical practice. IANB was performed using the device on five patients who had undergone detailed computed tomography examination for chronic orofacial pain in the third division of the trigeminal nerve. Lidocaine 1% (1 mL, no adrenaline added) was used as the local anesthetic. IANB was performed by three dentists with 2, 5, and 11 years of experience in orofacial pain treatment. Thus, the data were collected in triplicate for each patient. The primary endpoints were whether adjustment of the IANB device was required, changes in the sensation threshold of the lower lip, the time to disappearance of pain, the presence or absence of tongue sensation (“Do you have numbness in your tongue?”: “Yes/No”), and discomfort (visual analog scale). The incidence of any other adverse events was recorded. The procedure was judged to be successful if the pain disappeared and an elevation in the sensation threshold of the lower lip was observed. Adjustment of the IANB device was not required in any patient. A significant elevation in the sensation threshold of the lower lip and the disappearance of pain were observed in all patients. Three of the five patients reported experiencing tongue numbness. Discomfort with the use of the IANB device was less than 30 mm on the visual analog scale. No notable complications were observed. The appropriate type, concentration, and dosage of the local anesthetic must be considered during general dental treatment and oral surgical procedures. Our findings suggest that the IANB device is useful for eliminating errors between operators, enhancing safety, and improving the success rate.

## 1. Introduction

The inferior alveolar nerve block (IANB) is a useful nerve block in dental practice that can be utilized in various procedures, such as tooth extraction, oral implants, and pulpectomy, among other applications. IANB has a success rate of approximately 60–80%, indicating that failure of IANB may occur in some cases [1]. Failure of IANB can be attributed to the complex involvement of operator, anatomical, and environmental factors (e.g., tissue pH) [2]. Several complications associated with IANB, such as pain and trismus caused by needle insertion or withdrawal, facial nerve palsy, hematoma, blepharoptosis, external ophthalmoplegia, diplopia, and abducens nerve palsy, have been reported previously [1]. In addition, IANB may also result in nerve injury [3, 4] and local anesthesia poisoning [5]; therefore, some dentists prefer not to use IANB. However, IANB is a useful technique for pain control, and its reliable response offers significant benefits to operators and patients. Many dentists perform IANB by predicting the pterygomandibular space using anatomical markers. The pterygomandibular space includes the branches of the mandibular nerve, such as the lingual and inferior alveolar nerves, as well as the maxillary artery and the pterygoid venous plexus. These structures may be damaged by the needle tip during the conventional procedure of IANB, resulting in the aforementioned complications. Hence, the preoperative anatomical assessment of individual patients is the first step toward a safe and secure IANB. The first author previously developed and tested an IANB device for a skull model in a pilot study [6]. Although the pilot study verified the accuracy of the IANB device, its clinical effects and complications were not examined. This case series study aimed to use the “IANB device” in clinical practice to investigate its efficacy and the incidence of adverse events.

## 2. Materials and Methods

### 2.1. Participants

Patients who visited the Pain Clinic of the Tokyo Dental College Suidobashi Hospital between April 2020 and April 2022 and met the following inclusion criteria were included in this case series study for chronic orofacial pain: those who had undergone detailed computed tomography (CT) examination for chronic orofacial pain in the third division of the trigeminal nerve and those who had received IANB at least once a month for membranous pain, chronic osteitis, or other pain persisting for more than 6 months without sensory disturbance. As subjecting the patients to additional radiation exposure was unsuitable due to ethical considerations, only those who had undergone CT imaging of the mandible were included in this study. Only the CT images that included the mandibular dentition and mandibular lingula in the imaging range were selected. Patients who had not undergone CT imaging, minors (under 18), and those with sensory impairment in the third division of the trigeminal nerve were excluded. Informed consent was obtained from the participants using the research explanatory documents and consent form approved by the Ethics Review Committee of the Tokyo Dental College (approval number: 1068).

### 2.2. Creation of the IANB Device

**Figure 1:**
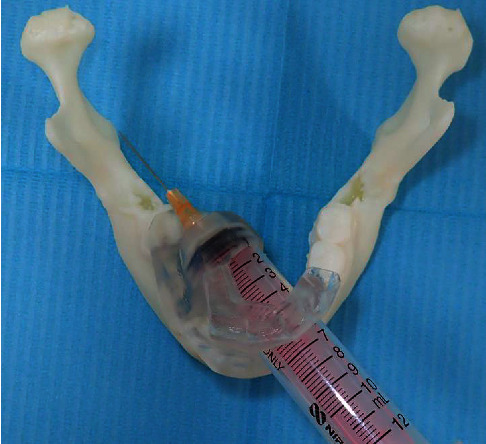
IANB device used in this study. This simulation of the IANB device using a syringe shows that the needle correctly reached the target point (mandibular lingula). IANB: inferior alveolar nerve block.

The creation and design of the IANB device followed the procedures described previously [6]. The dentition of each participant was optically scanned using TRIOS 3 (3 shape, Poland), and the obtained dentition data were matched with existing CT data of the mandible using computer-aided design (CAD) software (Materialize Magics™, Materialize, Leuven, Belgium). The IANB device was designed and printed with a 3D printer (Objet 260 connex™, Stratasys, Eden Prairie, USA). Biocompatible resin MED610 (Stratasys, Eden Prairie, USA) and supporting material SUP705B (Polymerized™, Stratasys, Eden Prairie, USA) were used. As part of the design, a safety margin was created by providing a guide and stopper with a target point 5 mm in front of the mandibular lingula (Figure 1). If the IANB device was not placed in the correct position, the intraoral scanning process was repeated to create the device again.

### 2.3. Local Anesthetic

Lidocaine 1% (1 mL, with no adrenaline; Xylocaine™ Injection Polyamp, Sandoz K.K., Tokyo, Japan) was used as the local anesthetic and injected using a 10-mL disposable syringe (Terumo, Tokyo, Japan) with a 25-gauge/25-mm injection needle (Terumo, Tokyo, Japan).

### 2.4. Procedure

The oral cavity of each participant was disinfected using a 30-fold diluted solution of povidone-iodine to disinfect the injection site, and the stable and correct installation of the IANB device was confirmed. Subsequently, the syringe was advanced to the stopper, and the local anesthetic was slowly injected after confirming the absence of numbness in the tongue and aspiration of blood. Measurements were taken upon the completion of the injection, and the effect was evaluated. IANB was performed by three dentists with 2, 5, and 11 years of experience in orofacial pain treatment. Thus, the data were collected in triplicate for each patient. Data were collected at intervals of at least 30 days.

### 2.5. Evaluation

The requirement for the adjustment of the IANB device was recorded. As the accuracy of the IANB is affected by the suitability of the dentition, a new IANB device was created for participants requiring adjustment.

**Figure 2:**
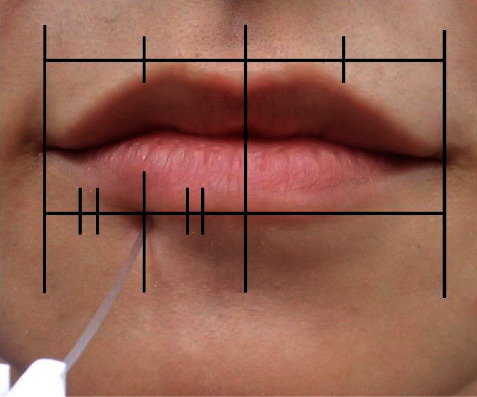
Evaluation site examined using a Semmes-Weinstein monofilament. The side of the lower lip on which the IANB was performed was further bisected at the midpoint, and the transitional area between the vermilion and skin at this point was set as an evaluation site. IANB: inferior alveolar nerve block.

We examined the changes in the sensation threshold of the lower lip as an objective evaluation. To determine the effect, the side of the lower lip on which the IANB was performed was further bisected at the midpoint, and the transitional area between the vermilion and skin at this point was marked as the evaluation site. Sensation at the evaluation site was determined using Semmes-Weinstein (SW) monofilament (SOT-DM20A™, SAKAI Medical, Tokyo, Japan) (Figure 2).

An SW monofilament was slowly brought into contact with the evaluation site, held for 2 s while slightly bent, and then slowly released for evaluation. Sensation before IANB was evaluated by asking the participants, “Can you feel it being touched?” Sensation after performing IANB was evaluated by asking the participants, “Has the pain disappeared?” every 30 s after the injection of the local anesthetic. When the participants no longer perceived the SW monofilament, the weight (g) was increased stepwise to higher values, and the perceptible weight (g) was recorded. This effect was evaluated for 600 s. The time to the disappearance of pain was measured as a subjective sensation. The procedure was judged to be successful if the pain disappeared and an elevation in the sensation threshold of the lower lip was observed; an inability to achieve these was judged as failure of IANB. The presence or absence of tongue sensation was assessed after the evaluation of lower lip sensation by asking the participants, “Do you have numbness in your tongue?” (“Yes/No”). Lastly, discomfort during the use of the IANB device was evaluated using the visual analog scale (VAS). The assessment was performed orally rather than using self-administered questionnaires. The incidence of any complications or discomfort was recorded.

### 2.6. Statistical Analysis

The effect of the IANB was determined by testing the difference in the weight (g) of the SW monofilament measured every 30 s. The Shapiro–Wilk test was performed for normality testing. Mauchly's sphericity test was performed for parametric data. If the assumption of sphericity was met, repeated measures analysis of variance (ANOVA) was selected; otherwise, the Greenhouse–Geisser corrected *P* value was adopted. The Friedman test was used for nonparametric data. In addition, post hoc comparisons were performed to determine the presence of differences between the measurement times. The adjusted significance level was set at *p* < 0.05. The Statistical Package for the Social Sciences (SPSS) version 24 statistical package (International Business Machines Corp., Armonk, NY, USA) was used for statistical analysis.

## 3. Results

### 3.1. Participants

Five patients were eligible for participation in this study. None of the patients met the exclusion criteria. Therefore, the study included all five participants, and 15 data collections were performed (three per patient). Although the study design was not exclusive to women, all participants included were females with an average age of 60.8 ± 4.1 years. The mean graded chronic pain scale version 2.0 score at consent for the study was 1.6. Four participants were diagnosed with posttraumatic trigeminal neuropathy (two participants had postextraction pain and two participants had postosteomyelitis pain), and one patient was diagnosed with neuralgia of an unknown cause.

### 3.2. Presence or Absence of Device Adjustment

None of the participants required adjustment of the IANB device.

### 3.3. Changes in the Sensation Threshold of the Lower Lip

**Figure 3:**
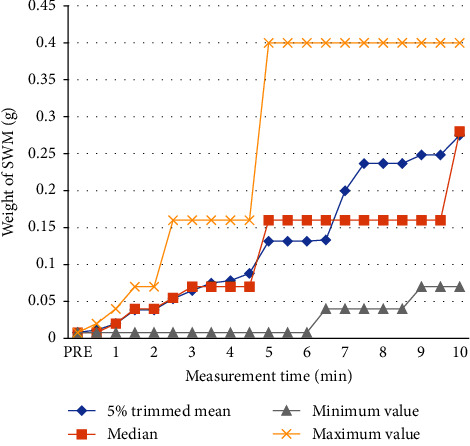
Distribution of the weight of SWM at each measurement time. The vertical axis indicates the weight (g) of the SWM and the horizontal axis indicates the measurement time (min). A rise in the perceptual threshold (SWM weight) can be observed over time. The increase in the median value is noticeable from 4 to 5 minutes. SWM: Semmes-Weinstein monofilament.

All participants showed elevation in the sensation threshold of the lower lip (Figure 3). The Friedman test was used as the distribution of the obtained data was nonparametric. A significant difference was observed in the weight (g) of the SW monofilament between the measurement times (*p* < 0.001). The total number was 15, the test statistic was 271.7, and the number of degrees of freedom was 20.

The Bonferroni method was used for post hoc comparisons to determine the presence of differences between the measurement times, and adjustments for the significance level were made. A significant difference was observed between the pre/30 s and all measurement times after 270 s, between 60 s and 300–600 s, between 90 s/120 s and 330–600 s, between 150 s and 420–600 s, and between 180 s and 450–600 s. The measurement times after 210 s showed no significant difference (Table 1).

### 3.4. The Time to the Disappearance of Pain

Pain disappeared in all participants, and the time required for the disappearance of pain was 56.7 ± 26.3 seconds.

### 3.5. Presence or Absence of Tongue Sensation

Three of the five participants answered “Yes” to the question, “Do you have numbness in your tongue?” The result was similar for all three data collection points. Numbness of the tongue was observed in 9 out of 15 data collection points.

### 3.6. Discomfort

Discomfort (VAS) during the use of the IANB device was 20.7 ± 13.8 mm.

### 3.7. Complications

No notable complications occurred with the use of the IANB device.

## 4. Discussion

Conventional IANB is performed blindly; thus, its success rate varies depending on anatomical differences among patients and operator skill. The IANB device used in the present study can account for the anatomical factors of patients using CT evaluation and the technical factors of operators using the guide structure that directs the needle tip to the target point simulated in the software. Furthermore, the stopper structure placed 5 mm in front of the mandibular lingula creates a safety margin to prevent nerve injury. A key aspect of this study was the safety margin, as its position too close to the inferior alveolar nerve leads to an increased risk of nerve damage, whereas a position too far from the nerve results in ineffectiveness. The safety margin was set at 5 mm based on the average error of 0.63 mm in the pilot study, and this distance was thought to be sufficient for preventing nerve injury. Since the basic idea of IANB is to fill the pterygomandibular space with the local anesthetic and induce an anesthetic effect on the inferior alveolar nerve [3, 7], the distance need not be shorter. In addition, it was previously reported that there was no difference in the effect of the needle tip being close to or far away from the mandibular ramus [8]. The safety margin of 5 mm set in the protocol of this study was found to be sufficiently effective, with no reported complications. The results are discussed below.

### 4.1. Participants

From an ethical point of view, the participants of this study were patients who had chronic orofacial pain in the third division of the trigeminal nerve, underwent CT evaluation in advance, and frequently received IANB. Due to this strict condition of participant selection in the implementation environment of this study, we expected that the number of study participants would be small. Therefore, data were collected in triplicate for each individual in this study. Due to its stable effect, all participants wished to continue the use of the IANB device even after the completion of the study.

### 4.2. IANB Device

None of the participants required adjustment of the IANB device, and no adjustments were required in the pilot study, indicating the high accuracy of the protocol for creating the IANB device used in this study. In addition, as the target point was set at the mandibular lingula, there were concerns that the presence of the sphenomandibular ligament would have an adverse effect [9]. However, we obtained a high success rate, similar to a previous study targeting the mandibular lingula [10].

### 4.3. Local Anesthetic

Conventionally, lidocaine is used at a concentration of 2% and often at a dosage of 1.8 mL [11]. However, to examine the accuracy of the IANB device, lidocaine was used at a low concentration and dosage in this study. An increased dosage with a high concentration would make it easier to infiltrate the mandibular nerve, increasing the maximum efficiency [11]. However, we used 1 mL of 1% lidocaine, considering the reliability of the IANB device would increase if the effect onset was achieved at a lower concentration and dosage. If secure IANB can be achieved at this concentration and dosage, it will contribute to a decrease in toxicity to the mandibular nerve [4] in patients receiving IANB frequently.

### 4.4. Changes in the Sensation Threshold of the Lower Lip

The effect of the IANB was determined using the weight (g) of the SW monofilament in this study. Although some studies have used methods involving an electric pulp test [11–15], we excluded invasive tests due to ethical considerations. In addition, our method is advantageous in that tactile sensation can be examined precisely at each measurement time. An elevated sensation threshold of the lower lip was observed in all participants. Furthermore, all measurement times after 270 s showed significant differences from the values before the IANB. This suggests that it may be necessary to wait until 270 s for effect onset when performing IANB with the IANB device in dental treatment, such as tooth extraction or pulpectomy. Previous reports have indicated that numbness of the lower lip appears in approximately 4.5–6 minutes in successful IANB and response in the pulp appears after approximately 5–20 minutes [8, 15, 16]; this suggests the time of effect onset in IANB with the IANB device is comparable with that in conventional IANB. An effect-onset delay, in which the effect appears after 15 min [11, 17], is said to occur in 12–20% [11, 14]. However, all participants in the present study showed effect onset within 10 min, and no effect-onset delay was observed. There have been attempts to increase the dosage of local anesthesia as a countermeasure against effect-onset delay and failure; however, IANB in the present study was performed successfully at a dosage of 1 mL, which is less than that used in the conventional method, without any effect-onset delay. This finding indicates that as the use of the IANB device allows for direct injection of local anesthetic near the mandibular foramen, the short infiltration distance of the local anesthetic leads to the achievement of a more secure IANB than the conventional method. The effect onset is expected to change depending on the type [14, 16, 18], concentration, and dosage [11] of the local anesthetic, and additional studies are needed.

### 4.5. The Time to the Disappearance of Pain

The time required for the participants to notice the disappearance of pain was 56.7 s. As pain in patients with chronic orofacial pain [19] is not purely nociceptive, its clinical significance differs from that of IANB required in general dental treatment. Nevertheless, we observed awareness of the effect within 1 min. At the institution where this study was conducted, IANB is often used as a treatment for peripheral sensitization in patients with chronic pain; however, patients have often pointed out its inconsistent or lack of effect and errors between operators. Clinical application of the IANB device yielded a constant effect and eliminated errors between operators, suggesting that it will contribute to the improvement of such treatment environments for patients with chronic orofacial pain.

### 4.6. Presence or Absence of Tongue Numbness

Three of the five participants answered “Yes” to the question, “Do you have numbness in your tongue?” Tongue numbness indicates infiltration of the local anesthetic into the lingual nerve [20]. The lingual nerve is located more anteromedially than the inferior alveolar nerve, and the IANB device with a target point 5 mm in front of the mandibular lingula could have caused a high probability of lingual nerve block. Since there is no need for a lingual nerve block except for the indication of glossalgia, the design may need to be reviewed.

### 4.7. Discomfort

The VAS during the use of the IANB device was 30 mm or less, indicating no major discomfort. Although the local anesthetic was injected slowly in this study, the injection speed may have affected the level of discomfort [21]. In addition, the use of the IANB device eliminated shaking of the needle tip, which likely reduced discomfort during needle insertion.

### 4.8. Complications

Although various complications of IANB, such as local anesthesia poisoning and nerve damage, have been reported [3], no notable complications occurred in the present study. Local anesthesia poisoning can be prevented by aspiration testing prior to injection; however, it is difficult to prevent nerve damage with conventional blind IANB [4]. Preoperative anatomical evaluation using CT [7] is important, and since the target point was set 5 mm in front of the mandibular lingula, the use of the IANB device can at least prevent injury to the mandibular nerve immediately before the inferior alveolar nerve. It is difficult to evaluate the lingual nerve using CT as it resides in soft tissue. However, in this study, the lingual nerve was expected to be in a loosened and flexed state as the participants were biting on the IANB device. We believe that this maintained the distance from the mandibular lingula and prevented needle skewering. Moreover, as conventional IANB applies the needle tip to the inner surface of the mandibular ramus, the needle is inserted at different angles multiple times [22], and the needle tip hitting the bone surface causes it to deform like a fishhook [23], increasing the risk of nerve damage. However, this did not occur with the use of the IANB device. Such a safety-conscious design is thought to contribute to the peace of mind of both operators and patients.

### 4.9. Limitations

As this was a case series study, and the number of data points was extremely small. Thus, the results should be interpreted with caution.

## 5. Conclusions and Clinical Implications

This study was conducted on patients with chronic orofacial pain, and we confirmed the usefulness of the IANB device. However, as the onset time and duration of the effect are important for its use in general dental treatment and oral surgical procedures, the appropriate type, concentration, and dosage of local anesthetics need to be considered. The results of this study suggest that this IANB device is useful for eliminating errors between operators, enhancing safety, and improving the success rate when compared with those of the conventional method. To further establish the efficacy and safety of this IANB device, its application on a larger scale in fields other than orofacial pain is necessary.

## Figures and Tables

**Table 1:** Post hoc comparisons.

Post hoc comparison before and after IANB	Test statistic	Standard error	Standardized test statistic	Significance probability	^*∗*^Adjusted significance
Pre-30 s	−0.267	2.266	−0.118	0.906	1
Pre-60 s	−1.767	2.266	−0.78	0.436	1
Pre-90 s	−3.7	2.266	−1.633	0.102	1
Pre-120 s	−3.7	2.266	−1.633	0.102	1
Pre-150 s	−5.2	2.266	−2.295	0.022	1
Pre-180 s	−5.933	2.266	−2.619	0.009	1
Pre-210 s	−7.567	2.266	−3.34	<0.001	0.176
Pre-240 s	−8.033	2.266	−3.546	<0.001	0.082
Pre-270 s	−8.733	2.266	−3.855	<0.001	0.024
Pre-300 s	−11.8	2.266	−5.208	<0.001	<0.001
Pre-330 s	−12.6	2.266	−5.561	<0.001	<0.001
Pre-360 s	−13.2	2.266	−5.826	<0.001	<0.001
Pre-390 s	−13.2	2.266	−5.826	<0.001	<0.001
Pre-420 s	−14.1	2.266	−6.223	<0.001	<0.001
Pre-450 s	−15.067	2.266	−6.65	<0.001	<0.001
Pre-480 s	−15.067	2.266	−6.65	<0.001	<0.001
Pre-510 s	−15.067	2.266	−6.65	<0.001	<0.001
Pre-540 s	−15.067	2.266	−6.65	<0.001	<0.001
Pre-570 s	−15.067	2.266	−6.65	<0.001	<0.001
Pre-600 s	−15.067	2.266	−6.65	<0.001	<0.001

This table shows an excerpt of the differences between premeasurements and other measurement times. ^*∗*^The Bonferroni correction was used to adjust the significance values for multiple tests. IANB: inferior alveolar nerve block.

## Data Availability

All the data obtained from the research cannot be disclosed in the research protocol approved by the Tokyo Dental College Ethics Review Committee. Most of the data needed to interpret the study results are provided in the text.
